# Susceptibility of Human Spermatozoa to Titanium Dioxide Nanoparticles: Evaluation of DNA Damage and Biomarkers

**DOI:** 10.3390/life14111455

**Published:** 2024-11-09

**Authors:** Elena Maria Scalisi, Roberta Pecoraro, Agata Scalisi, Jessica Dragotto, Giovanni Bracchitta, Massimo Zimbone, Giuliana Impellizzeri, Maria Violetta Brundo

**Affiliations:** 1 Department of Biological, Geological and Environmental Sciences, University of Catania, 95124 Catania, Italy; roberta.pecoraro@unict.it (R.P.); scaliagata@gmail.com (A.S.); mariavioletta.brundo@unict.it (M.V.B.); 2 U.O. Fisiopatologia della Riproduzione Umana—Clinica del Mediterraneo, 97100 Ragusa, Italy; jessica.dragotto@gmail.com (J.D.); bracchitta@centroaster.com (G.B.); 3 CNR-IMM, 95123 Catania, Italy; massimo.zimbone@ct.infn.it (M.Z.); giuliana.impellizzeri@ct.infn.it (G.I.)

**Keywords:** sperm, TiO_2_-NPs, SHBG, DNA fragmentation, biomarker stress, endocrine disruptor

## Abstract

Nowadays, developing countries have seen a reduction in male reproductive parameters, and it has been linked to the exposure of endocrine disrupting chemicals (EDCs), which are able to mimic or disrupt steroid hormone actions. Also, nanoparticles have shown effects on the male reproductive system, in particular the use of TiO_2_-NPs in drugs, cosmetics, and food as pigment additives, and, thanks to their small size (1–100 nm), provide themselves the opportunity to be internalized by the body and pass the blood–testis barrier (BTB). Therefore, TiO_2_-NPs can act on spermatogenesis and spermatozoa. In this study, we carried out an *in vitro* assay on human spermatozoa to evaluate the effects of TiO_2_-NPs at the concentrations of 500, 250, 100, and 50 ppm. Exposure did not statistically alter sperm parameters (e.g., motility and viability) but induced damage to sperm DNA and the expression of biomarkers by spermatozoa. This immunofluorescence investigation showed a positivity for biomarkers of stress (HSP70 and MTs) on the connecting piece of spermatozoa and also for sex hormone binding globulin (SHBG) biomarkers. The SHBG protein acts as a carrier of androgens and estrogens, regulating their bioavailability; therefore, its expression in the *in vitro* assay did not rule out the ability of TiO_2_-NPs to act as endocrine disruptors.

## 1. Introduction

The declining birth rate is, today, the most serious social problem among the industrialized countries [1,2]. Daily human exposure to environmental toxic pollutants, such as pesticides and herbicides [3,4,5], heavy metals [6,7,8], and plastics compounds [9,10,11], have resulted in negative effects on the male reproductive system [12,13] with consequent damage to the functionality of the testicles. As demonstrated by *in vivo* studies, the pollutants cause lower semen concentration, lower total sperm count, lower sperm motility, and abnormal morphology on tail and head sperm; moreover, they are able to bind/block androgen receptors and alter hormone/receptor metabolism, testosterone production, and Leydig and Sertoli cell functions [14,15,16,17]. It seems clear that they are molecules able to mimic or disrupt steroid hormone actions [18]; for this reason, they are called “endocrine disruptors”, and exposure to them can occur during professional activities or by accidentally using everyday objects that contain them. Similar to these pollutants, nanoparticles also produce the following results: several studies in laboratory animals reported the ability of metal-based NPs to change the structure of testes, which causes an alteration in seminiferous tubules, affecting their vacuolization; a reduction in the germinative layer’s thickness and lower cellular adhesion of seminiferous epithelia; a reduction in the vacuolization of Sertoli cells [19,20,21,22,23,24,25]; and an alteration in the biosynthetic and catabolic pathways of hormones [20,26,27,28]. This implies a reduction in sperm density and motility [16,17,29,30], an increase in sperm abnormality [31,32], and DNA damage, which can negatively affect reproductive success. Among metal oxide nanoparticles, the toxicity of titanium dioxide nanoparticles (TiO_2_-NPs) has been investigated because they are the most used thanks to their multiple applications [33]. TiO_2_-NPs find applications in electronics, biomedicine, pharmaceuticals, cosmetics, the food industry as pigment additives for their brightness (gum, candy, puddings), in paints for their opacifying strength (hiding power), and in wastewater treatments as a photocatalyst to remove contaminants thanks to their ability to absorb UV light [34]. As a result of the various applications of TiO_2_-NPs, the investigations into its toxicity have increased [35,36] because, for example, in food, TiO_2_-NPs can be used as an additive without any upper limit (*quantum satis*), as long as they are used in accordance with the Good Manufacturing Practice (GMP) [37]. In contrast, the use of TiO_2_-NPs as additives in pharmaceutical products (gelatine capsules, tablet coatings, and syrups) [38] is not regulated, so the concentrations of TiO_2_-NPs to which consumers are exposed are not specified and can increase with drug consumption. A bioaccumulation of TiO_2_-NPs in the body causes adverse health effects, as suggested by several studies [39]. In the male reproductive system, oral administration of TiO_2_-NPs can cause a change in the relative weight of testes and accessory male sex organs [40], and within the testes, TiO_2_-NPs can cause a reduction in the number of germ cells, spherospermia, vacuoles in the interstitial glands, and vacuolization of spermatogenic cells [41].

Because of their small size, TiO_2_-NPs can cross the circulatory system and arrive at male gonads, affecting the testicular structure as mentioned. The effects of TiO_2_-NPs on spermatozoa are poorly investigated, even if the exposure to them may occur during their use. The TiO_2_-NPs are able to produce reactive oxygen species (ROS), causing oxidative stress [42]; therefore, in our study, we investigate the susceptibility of spermatozoa to different concentrations of TiO_2_-NPs, evaluating the expression of markers of stress (HSP70 and MTs) and DNA damage. Moreover, to point out the action of TiO_2_-NPs as endocrine disruptors, we evaluate the marker, sex hormone binding globulin (SHBG), which is a protein that binds to androgens with high affinity and specificity but can be influenced by endocrine disruptors. Also, the sperm parameters, namely, motility and viability, are evaluated. Nanoparticles are preventable risk factors; thus, an accurate characterization of their action on spermatozoa could improve understanding of reproductive risks.

## 2. Materials and Methods

### 2.1. Preparation and Dosage of Titanium Dioxide NPs

Powders of the titanium dioxide nanoparticle (Degussa, P25) supplied by CNR-IMM (Microelectronics and Microsystems of Catania-National Research Council, Italy) were purchased from Sigma Aldrich (St. Louis, MO, USA). According to the protocol procedure of Zimbone [43], the hydrodynamic diameter was measured by dynamic light scattering (DLS) to characterize the nanoparticle solution. Due to the lack of indications about TiO_2_-NPs concentration in drugs, four concentrations (50, 100, 250, and 500 ppm) were selected. All working solutions were prepared by dispersing the powders of TiO_2_-NPs in Sperm Medium Gems^®^ (Genea Biomedx, Sydney, Australia) with 4 cycles of sonication for 10 and 5 min of breaks, using a probe sonicator (Bandelin Sonopuls) (BANDELIN electronic GmbH & Co. KG, Berlin, Germany) to ensure the homogeneous dispersion of titanium dioxide nanoparticles [44].

### 2.2. Spermatozoa Sample Collection and Protocol Procedures

#### 2.2.1. Exposure Procedure

For the research, sperm samples were obtained from healthy and fertile men (between 20 and 39 years of age) recruited from the fertility center MEDI.SAN “Clinica del Mediterraneo di Ragusa/Centro ASTER” (RG). In addition, the Ethics Committee of the Center of P.M.A. (Procreazione Medicalmente Assistita) MEDI.SAN “Clinica del Mediterraneo di Ragusa/Centro ASTER” (RG) approved the research with the following reference number: 190002.

Before sperm collection, 3–5 days of abstinence were recommended and all subjects signed an informed consent according to the guidelines established for research on humans by the Declaration of Helsinki [45]. Especially, we selected samples whose semen parameter satisfied the values reported in Table 1, as suggested by the WHO laboratory manual (2021) [46].

All the experiments were performed according to good laboratory practice by WHO guidelines 2021 and Organization for Economic Cooperation and Development (OECD) guideline [46,47].

The collected sperm samples (*n* = 10) were incubated at 37 °C to ensure their liquidation within 60 min. Then, they were divided into aliquots (5 × 10^6^ spermatozoa/mL) and mixed (1:1 ratio) with Sperm Wash Medium^®^ (Genea Biomedx, Sydney, Australia) to separate the spermatozoa from the seminal fluid by centrifugation (10 min at 1500 rpm). The supernatant was discarded and each pellet was treated with 500 μL of TiO_2_-NPs working solution. Sperm aliquots were prepared for treatment with the 50, 100, 250, and 500 ppm TiO_2_-NPs working solutions, including a control, in which the sperm pellet was incubated only with sperm medium.

The exposure for all samples was set for 1 h at 37 °C in a cell culture incubator (Celis) (RIEGER Industrievertretungen GmbH, Vienna, Austria) with 5% CO_2_ and the Eppendorf tube inclined of 45° and the cap open. At the end of exposure for all samples, including the control sample, we analyzed the supernatants containing motile-selected spermatozoa [35] to evaluate their motility, viability, DNA damage, oxidative stress, and expression of biomarkers (HSP70, MT, SHBG). All the experiments were performed in triplicate, and 3 replicated slides were analyzed for each experiment.

#### 2.2.2. Assessment of Sperm Motility and Viability

At the end of exposure, first of all, we assessed the sperm motility using an optical microscope (Set E200 Nikon, Tokyo, Japan) at 400× magnification. For each TiO_2_-NPs-exposed group and also for the control group, 10 μL of sample was placed on a slide [47] in order to identify motile spermatozoa (with different degrees of motility) from immotile ones. To calculate the percentage of progressive, nonprogressive, and immotile spermatozoa, a number of 100 cells (spermatozoa) for slides were counted.

The eosin test was used to assess the number of viable cells. This method is based on the dye exclusion principle: if the cell membrane is damaged, eosin penetrates and dead sperms will appear red-stained; if the cell membrane is intact, eosin cannot enter, and living sperm will be unstained [47]. A mixture of 10 μL of semen sample and 10 μL of Eosin Y solution 0.5% (Bio-Optica, Milan, Italy) both for exposed groups and the control group was prepared and immediately observed by light microscope (Set E200 Nikon, Amsterdam, Netherlands) at 400×.

Also, for viability, a number of 100 spermatozoa for slides were counted in order to distinguish living cells from dead ones (respectively, not stained and red-stained).

#### 2.2.3. Scanning Electron Microscopy

The presence of TiO_2_-NPs on sperm cell membrane was evaluated using scanning electron microscopy. Semen samples underwent centrifugation (2000 rpm, 3 min) and were then fixed in glutaraldehyde (2.5%) for 1 h at 4 °C. Once fixed, sperm pellets were washed twice for 5 min each in phosphate-buffered saline (PBS) and dehydrated gradually in ethanol series (from 35° up to 100°) for 2 min each.

Finally, we used a solution of alcohol 100° and hexamethyldisilazane (Merck KGaA, Darmstadt, Germany) in a 1:1 ratio and then only hexamethyldisilazane for 1 min. The pellets were placed on SEM stubs for gold sputter coating.

Three cycles of metallization, each of 1 min, were carried out using a metallizer instrument (Coxem, Daejeon, Republic of Korea) with a current of 5 mA. SEM–EDX analysis (Cambridge Stereoscan 360C Instruments, EDX, INCA, Oxford, UK) was carried out.

#### 2.2.4. Evaluation of Oxidative Stress

The oxidation–reduction potential (ORP) was measured by the MiOXSYS System in order to detect oxidative stress (OS). It directly evaluates the redox balance between ROS and antioxidants.

A total of 30 μL of semen sample was placed in the sample port of the sensor, and after a few minutes, the ORP value (expressed as millivolts) was displayed.

A normalized ORP value was calculated (raw ORP value was divided by the sperm concentration (mV/10^6^ sperm/mL)). A higher ORP value means a higher oxidant activity [48]. ORP value was expressed as a mean value for each experiment.

#### 2.2.5. Assessment of DNA Damage: Sperm Chromatin Dispersion Test (SCD) and TUNEL Assay

According to the sperm chromatin dispersion test (SCD), DNA dispersion halos will be produced by sperm nuclei without DNA fragmentation, while no halo will be present in sperm nuclei with DNA fragmentation [49]. These findings occur after an acid denaturation and removal of nuclear proteins of spermatozoa.

The sperm chromatin dispersion test was performed following the instructions provided by Halosperm^®^ kit (Halotech DNA, Madrid, Spain).

An aliquot of each sperm sample (25 μL) was carefully mixed with the agarose included in the kit (previously melted in a water bath at 90–100 °C for 5 min).

Then, 25 μL of the cellular suspensions were placed on slides, covered with coverslips (22 mm × 22 mm) and left at 4 °C for 5 min.

As the gel was just produced with the embedded spermatozoa, the coverslip was removed with care and the denaturation solution (DA-solution) included in the kit was added for 7 min at RT.

Then, slides were placed into the lysing solution (Triton X-100, Dithiothreitol, Madrid, Spain) for 25 min and washed away using distilled H_2_O (5 min, RT).

After dehydration in 70%, 90%, and 95% ethanol, the slides were dried and stained by Diff-Quik for bright-field microscopy.

The slides were kept in the dark at RT, then observed under a light microscope (1000×).

The TUNEL is an *in situ* test used to study sperm DNA fragmentation. Thanks to the enzyme terminal deoxynucleotidyl transferase (TdT), modified deoxyuridine triphosphate (dUTPs) are incorporated at the 3′-OH end of fragmented DNA.

If there are any breaks in the DNA, they can be identified by released fluorescence, since dUTPs are conjugated with fluorescent dyes (fluorescein-dUTP).

Sperm cells (2 × 10^6^ sperm/mL) of each group were smeared on slides, fixed in 4% PFA (1 h), and permeabilized using 0.25% buffered Triton for 20 min at RT.

Finally, a washing was performed twice with deionized H_2_O before carrying out the TUNEL test following the instruction protocol (Click-iT^®^ Plus TUNEL kit, ThermoFisher Scientific, Waltham, MA, USA).

Observations were performed using a fluorescent microscope (Nikon ECLIPSE Ci fluorescence, Amsterdam, The Netherlands), counting 200 spermatozoa, as recommended by WHO [47], and photos were taken through a Nikon DS-Qi2 camera connected to the microscope.

#### 2.2.6. Marker Identification on Spermatozoa

To evaluate the effects of TiO_2_-NPs on spermatozoa, biomarkers of oxidative stress, namely, metallothioneins (MTs) and heat shock protein (HSP70), were investigated in all experimental groups and compared with the control group. Moreover, sex hormone binding globulin (SHBG) was investigated as a biomarker to highlight the action of TiO_2_-NPs as endocrine disruptors.

At the end of the exposure, all sperm samples (treated and control groups) were diluted in PBS and adjusted to 2 × 10^6^ cells/mL, to be fixed at RT for 10 min with 1% glutaraldehyde phosphate.

The fixative was removed by centrifugation at 2000× *g* for 10 min and the pellet was washed in PBS twice for 5 min. Subsequently, 40 μL of the cell suspension was placed on polylisined slides (DakoAgilent, Santa Clara, CA, USA) to perform the protocol steps in a humid chamber: permeabilization of spermatozoa with 0.5% (*v*/*v*) Triton X-100 in PBS for 15 min, washing with PBS (twice), and addition of 5% blocking solution (BSA) for 20 min to avoid nonspecific binding of antibodies. It was followed by incubation with primary antibody anti-mouse-MT (GeneTex, 1:1000), anti-mouse-HSP70 (GeneTex, 1:1000), and anti-rabbit- SHBG (GeneTex, 2 µg/mL, Irvine, CA, USA) at 4 °C overnight.

Once the incubation of the primary antibody was complete, the slides were washed three times to remove excess primary antibody using PBS; thus, the secondary antibody was added for 1 h at 4 °C. FITC-conjugated anti-mouse secondary antibodies (GeneTex, at a dilution of 1:1000) for HSP70 and MT, and TRITC-conjugated anti-rabbit secondary antibodies (GeneTex, at a dilution of 1:1000) for SHBG were used. Finally, the slides were washed again in PBS, and the spermatozoa nuclei were counterstained with medium containing DAPI (Abcam, Cambridge, UK). Rubber cement was used to seal the slide in order to preserve the fluorescence of the cells. Green fluorescence was observed by FITC-conjugated anti-mouse secondary antibody, while red fluorescence was observed by TRITC-conjugated anti-rabbit secondary antibody. Observations were performed with a Nikon ECLIPSE Ci fluorescence microscope. All samples were analyzed in duplicate and at least 100 spermatozoa were analyzed per slide.

#### 2.2.7. Assessment of Acrosomal Membrane Integrity

To assess the integrity of the sperm acrosomal vesicle, the fluorescently conjugated *Arachis hypogea* lectin (PNA) was used [50,51]. The pellet was fixed with glutaraldehyde (2.5%) for 20 min. Subsequently, we carried out our protocol procedure for incubation with 10 µg/mL PNA FITC-conjugated (Vector Laboratories, Newark, CA, USA), and all slides were observed under a fluorescence microscope [52] (Nikon ECLIPSE Ci fluorescence, Amsterdam, Netherlands).

#### 2.2.8. Statistical Analysis

Statistical analysis was performed with the one-way analysis of variance (ANOVA) test followed by Tukey’s test to assess statistically significant differences between the exposed and the control groups. Before performing the ANOVA analysis, the normal distribution of the data was verified with the Shapiro–Wilk test. The Past 4.0 software was used for statistical analysis, and a *p* < 0.05 was considered a statistically significant difference. All data are presented as mean ± standard deviation (SD).

## 3. Results

### 3.1. Characteristics of TiO_2_-NPs

**Figure 1:**
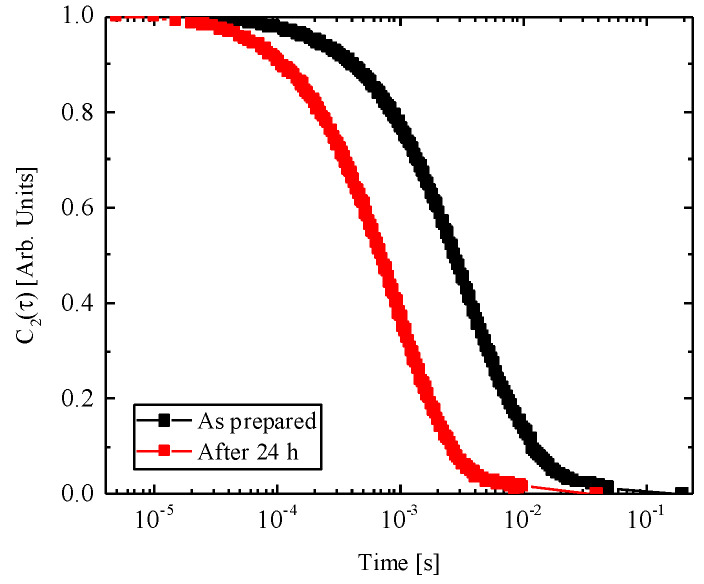
Autocorrelation function of the colloidal solution for both the “as prepared” solution (black curve) and “after 24 h” of sedimentation (red curve).

The nanopowders of TiO_2_ showed a crystalline phase mix of anatase (86%) and rutile (14%) and an average size of about 50 nm. The DLS autocorrelation function for “as prepared” solution and for solution after 24 h of sedimentation is shown in Figure 1. The hydrodynamic diameter is about 1100 nm for the “as prepared” sol (black curve). The sol were allowed to sediment for 24 h, and correlation functions were measured again. The hydrodynamic diameter decreased to about 283 nm (red curve). This indicates that the larger particles were deposited and only smaller aggregates remained in suspension during the experiment. Moreover, the TiO_2_-NPs have a pH of 7 and they are lightly negatively charged.

### 3.2. Sperm Motility and Viability

No statistically significant changes in motility were observed in all exposed groups, and even with regard to viability, the percentage of dead spermatozoa did not increase compared to the control (Table 2).

### 3.3. Observation by Scanning Electron Microscopy

**Figure 2:**
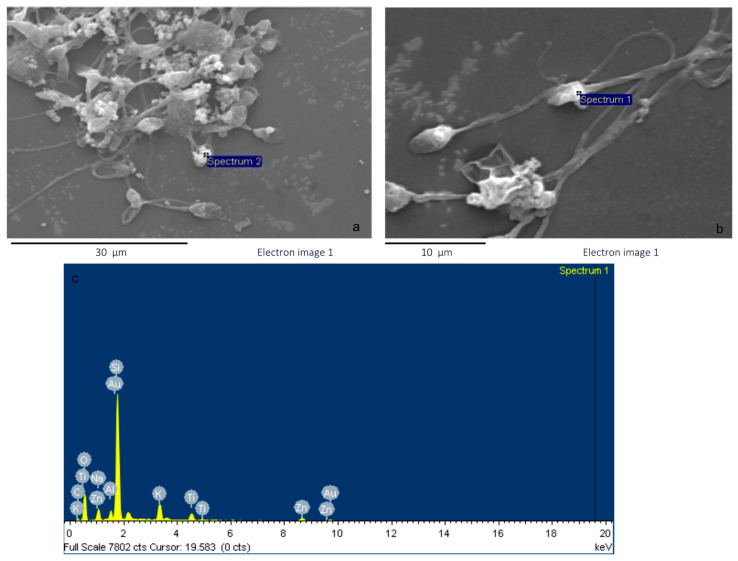
(**a**) TiO_2_-NPs on the sperm head exposed to 500 ppm TiO_2_-NPs. (**b**) Spermatozoa at higher magnification. (**c**) EDX energy-dispersive X-ray spectrum.

Scanning analysis showed that the TiO_2_-NPs are able to interact with the sperm plasma membrane, and, in particular, microanalysis demonstrated their localization on the sperm head, as shown in spermatozoa exposed to 500 ppm TiO_2_-NPs (Figure 2). Information about the elements found on the surface of spermatozoa are available in the Supplementary Materials (SI-1). The permanence of TiO_2_-NPs on the surface of spermatozoa can induce alterations of their membrane or lead to internalization of TiO_2_-NPs.

### 3.4. DNA Damage

**Figure 3:**
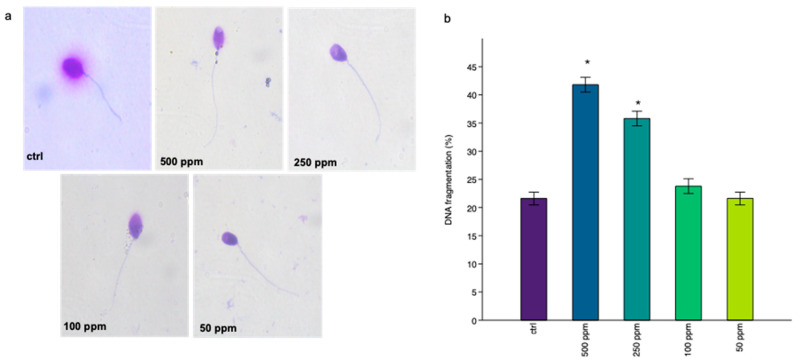
Observation of DNA fragmentation after exposure to TiO_2_-NPs by test (SCD). (**a**) Spermatozoon without fragmentation has a halo around the head, while spermatozoon with fragmentation has no halo around the head. (**b**) Data are shown as % average of spermatozoa with DNA fragmentation, and statistically significant differences are indicated with the symbol * (*p* < 0.05).

Thanks the sperm chromatin dispersion test (SCD), sperm nuclei with DNA fragmentation (absence of halo) were differentiated from sperm nuclei with intact DNA (presence of halo), as can be seen in Figure 3a. The average percentage of spermatozoa with fragmented DNA increased in all exposed groups compared to the control group; however, statistical analysis showed a significant increase in DNA fragmentation for spermatozoa exposed to 500 ppm and 250 ppm with a *p* < 0.05, indicated with the symbol * (Figure 3b).

Observation of slides according to the TUNEL protocol also confirmed DNA fragmentation in exposed groups. Slides observed under the fluorescent microscope showed a green fluorescence on spermatozoa with DNA breakage, because this was labeled on the free 3′-OH with a modified dUTP (fluorescein-dUTP) that carries a green fluorescence (SI-2).

### 3.5. Oxidative Stress

We observed that exposure to TiO_2_-NPs induces oxidative stress for all concentrations tested; the ORP value was significantly increased in the exposed groups compared to the control group (*p* < 0.05) (Table 3). This suggests that the antioxidant enzyme system of spermatozoa is not able to counteract the excessive presence of ROS, so they can cause an increase in the concentration of proteins involved in cellular stress.

### 3.6. Marker on Spermatozoa

**Figure 4:**
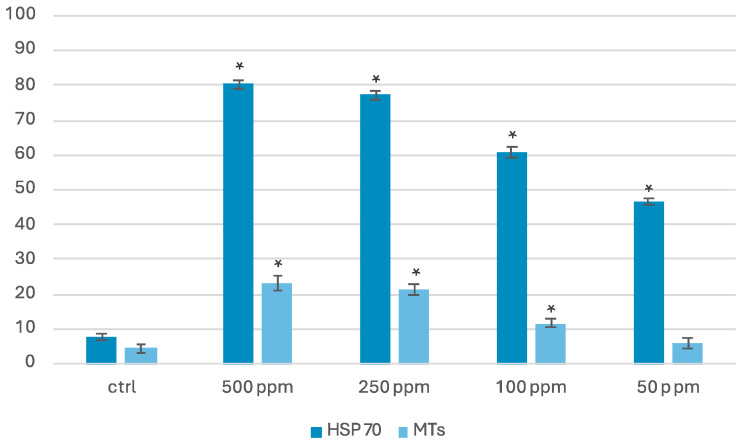
Mean percentage of spermatozoa that expressed HSP70 and MTs after the exposure of TiO_2_-NPs. For each experimental group, 100 spermatozoa were counted in five different fields. The asterisks (*) above the bars indicate statistically significant differences between the exposed groups and the control group (* *p* < 0.05).

Immunohistochemical investigation for biomarkers of oxidative stress, such as the metallothioneins (MTs) and the heat shock protein (HSP70), showed their expression on exposed TiO_2_-NPs samples. For both biomarkers, a positivity appeared on the connecting pieces of spermatozoa (SI-3). The average percentage of the spermatozoa that expressed HSP70 and MTs, at the end of exposure of TiO_2_-NPs, is shown in Figure 4. In all exposed samples, a higher percentage of positivity was found for the HSP70 biomarker as opposed to the MTs biomarker.

Statistical analysis performed for both biomarkers showed a statistically significant expression of the biomarker HSP70 in all exposed groups compared to the control, while for the MTs biomarker, a statistically significant expression was found in the 500 ppm, 250 ppm, and 100 ppm groups compared to the control group. A statistically significant difference (*p* < 0.05) between the exposed groups and the control group is indicated with the symbol *.

**Figure 5:**
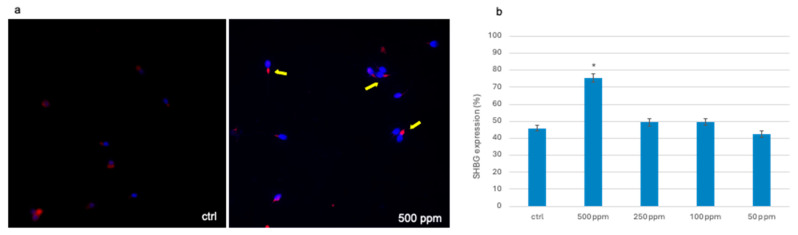
Localization of SHBG on spermatozoa treated with TiO_2_-NPs at the end of exposure, 1000× magnification. (**a**) Unexposed spermatozoa and spermatozoa exposed to 500 ppm TiO_2_-NPs. Nuclei blue (DAPI) and red fluorescence of SHBG protein is indicated by the yellow arrows. (**b**) Mean percentage of spermatozoa that expressed SHBG. Statistically significant difference is found at *p* < 0.05 (*).

At the end of the exposure, we also investigated the expression of the SHBG biomarker in order to point out the ability of TiO_2_-NPs to act as endocrine disruptors. Observation of the slides under a fluorescence microscope showed a positivity for SHBG in all exposed groups (SI-4); however, statistical analysis revealed a statistically significant difference between the exposed group to 500 ppm and control group, with a *p* < 0.05 (*). Figure 5a shows the positivity for SHBG on the connecting pieces of spermatozoa at 500 ppm compared to the control at 1000× magnification, while the average percentage of the spermatozoa that expressed SHBG is shown in Figure 5b.

The recorded semen parameters are summarized in Text SI-5.

### 3.7. Acrosome Damage

**Figure 6:**
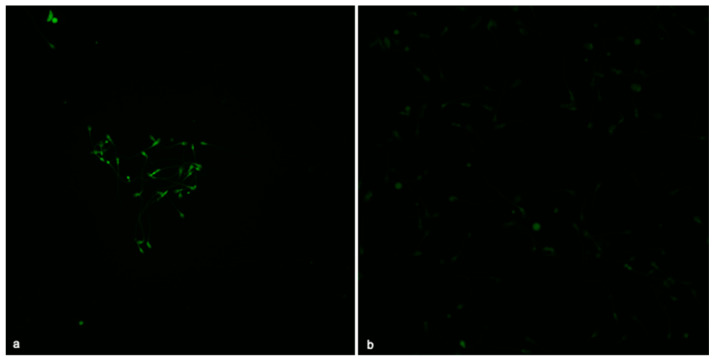
Acrosome integrity, 400× magnification. (**a**) Unexposed spermatozoa with positivity for PNA-lectin. (**b**) Spermatozoa exposed to 500 ppm TiO_2_-NPs, without positivity for PNA-lectin.

Finally, considering the results of ORP values and expression of oxidative stress biomarkers, we evaluated the integrity of the acrosome vesicle in all exposed groups. Thanks to the PNA-lectin protocol, no significant alteration of the acrosome vesicle was detected for the concentrations of 250, 100, and 50 ppm (SI-6), whereas statistically significant values were observed for the concentration of 500 ppm, compared to the control group, with *p* < 0.05 indicated with the symbol *. Figure 6 shows that in the group exposed to 500 ppm, there is an absence of positivity for PNA-lectin, suggesting a loss of the acrosome, while in the control group, an intact acrosome was observed, as suggested by the PNA-lectin positivity.

## 4. Discussion

*In vitro* assays are useful to identify and understand the toxicological effects of different molecules [53,54]; for example, the evaluation of nanoparticles (NPs) is usually based on *in vitro* cell assays to predict their toxicity before subjecting them to animals [55]. The male reproductive system, and thus the testes, appeared to be the main target of TiO_2_-NPs [56,57]; in particular, a decline in sperm parameters such as number, viability, abnormalities, and motility of spermatozoa has been reported with increasing concentrations of NPs by *in vivo* studies [40,41]. Despite this, investigations of the effect of TiO_2_-NPs on human spermatozoa by *in vitro* assay are poor, and sperm motility is a reliable predictor for sperm quality and fertilization success [58]. Our results showed that sperm motility was not impaired by NPs, nor was the viability of spermatozoa significantly decreased in exposed groups compared to control. However, as reported in the literature, the cytotoxic effect of NPs should not be underestimated, because NPs are able to interact with DNA, mitochondria, and various proteins [59,60,61,62,63]. In our study, although the sperm quality values were not altered, we found DNA damage, especially at the higher concentrations of TiO_2_-NPs, as suggested by the results of SCD and TUNNEL tests. This is in agreement with data for metal oxide nanoparticles [63,64,65]; in addition, Santonastaso and colleagues [63] highlighted genotoxic effects with loss of sperm DNA integrity in human spermatozoa *in vitro*. DNA damage occurs through the induction of oxidative stress by nanoparticles. Physiologically, ROS are natural byproducts of the normal oxygen metabolism and they affect cell signaling and homeostasis [66] in spermatozoa; ROS are important signaling molecules due to their hyperactivation and acrosome reaction [67]. Nevertheless, ROS are generated in the cellular response to exogenous substances and bacteria [68]; for this reason, an excess of them from external inputs such as NPs can increase oxidative stress (OS), resulting in damaged DNA and apoptosis [69,70]. This increase in ROS also leads to early exocytosis of the acrosomal vesicle, as demonstrated by its loss in spermatozoa exposed toTiO_2_-NPs (500 ppm). Evidence has shown that environmental pollutants can stimulate the acrosome reaction in mammals [71,72] and ROS mediate the acrosomal reaction in sperm [73]; however, when they are overgenerated, oxidative stress occurs and the antioxidant defense mechanism is not sufficient. For this reason, the cells can maintain cellular homeostasis under stress with the expression of HSPs [74]. HSPs are the main molecular chaperone proteins in eukaryotic cells [75], whose expression level changes under stressful condition such as hyperthermia, hypothermia, hypoxia, hyperoxia, oxidative stress, viral infection, and energy depletion [76,77]. Especially, the family of thermal shock proteins 70 (HSP70) is highly expressed on cells of the germinal epithelium [78,79], because it allows the recovery of DNA and RNA damage in the germinal epithelium [80]. The role of HSPs is to ensure progression and maintenance of sperm physical characteristics and function during normal fertilization [81] or after exposure of the sperm to different environmental challenges (thermal stress or freezing) [82,83]. HSPs have been identified on the surface of sperm membranes in bull, boar, mouse, rat, and human [84]. HSP70 is synthesized during spermatogenesis; therefore, the spermatozoa can respond to stress by consuming the pool HSP70 already synthesized with a redistribution of them [82,85,86]. This occurred in our study after exposure to TiO_2_-NPs. With regard to MTs, they have been identified in human male genital organs [87,88]. In rat testis, MTs have been observed in the seminiferous tubules containing differentiating spermatogenic cells [89]. Although the biological and physiological function of metallothioneins (MTs) in male genital organs is unclear, our results highlight that MTs maintain their biological role of detoxification from metals. Finally, the expression of the SHBG biomarker highlighted the ability of TiO_2_-NPs to act as endocrine disruptors. The homodimeric glycoprotein SHBG has a molecular mass of approximately 90 kDa [90], and it is well characterized in humans [91,92], where it is involved in the transport, regulation, and action of sex-steroid hormones. The main sex-steroid hormones that are linked by SHBG are testosterone, 5α-dihydrotestosterone, and 17β-estradiol; however, SHBG has shown affinity for synthetic steroids and also for environmental contaminants [93,94,95], which could alter the concentration of SHBG or induce its dysfunction with a consequent adverse reproductive effect, such as infertility [96]. Even if the SHBG is a circulating blood protein, it can also be produced locally in the brain, uterus, and testes [92,97]. In our previous investigation, an expression of SHBG by qRT-PCR was found in the cysts of the seminiferous tubules of the zebrafish testicle, where it improves spermatogenesis thanks the ability to bind the TiO_2_-NPs [98]. Similarly, in sperm, SHBG expression supports the ability of TiO_2_-NPs to act as endocrine disruptors. 

## 5. Conclusions

Fertility and successful reproduction depend on the quality of gametes which are important to sustain species. Although in our investigation the parameters of viability and motility were not so much altered by the exposure to TiO_2_-NPs, a fragmentation of spermatic DNA was observed at all tested concentrations. Moreover, the expression of the SHBG protein corresponding to the androgen binding protein (ABP) suggests that, like ABP, its increase could depend on xenobiotics. At the same time, spermatozoa are able to respond to the presence of TiO_2_-NPs with the expression of stress biomarkers (Hsp70 and MTs). This can certainly depend on the exposure time considered as well as on the shape and size of the nanoparticles. Overall, our results provide additional information to the data in the literature, because the effect of TiO_2_-NPs on spermatozoa through the expression of biomarkers (HSP70, MTs, SHBG) was studied, whereas previous studies have mainly focused on the action of TiO_2_-NPs on embryo development and the reproductive system through in *vivo* investigation.

## Figures and Tables

**Table 1:** Lower fifth percentile (with 95% confidence interval) of semen parameters from men in couples starting a pregnancy within one year of unprotected sexual intercourse leading to a natural conception, by WHO 2021.

Sperm Parameters	5th Percentile	(95% Confidence Interval)
Semen volume (mL)	1.4	(1.3–1.5)
Sperm concentration (10^6^ per mL)	16	(15–18)
Progressive motility (PR, %)	30	(29–31)
Nonprogressive motility (NR, %)	1	(1–1)
Immotile spermatozoa (IM, %)	20	(19–20)
Viability (%)	54	(50–56)

**Table 2:** Sperm viability and motility (n = 10 samples) after exposure to TiO_2_-NPs (the values are expressed as mean ± SD, and no statistical significance between exposed groups and the control group was found (*p* > 0.05)).

TiO_2_-NPs Concentration	Motility	Viability
500 ppm	82 ± 10	80 ± 15
250 ppm	78 ± 11	81 ± 13
100 ppm	79 ± 7	87 ± 6
50 ppm	68 ± 14	82 ± 10

**Table 3:** ORP values. The data are shown as mean ± standard deviation. Statistically significant differences are found between the control group and exposed groups (*p* < 0.05).

	ctrl	500 ppm	250 ppm	100 ppm	50 ppm
sORP (mV)	177.09 ± 9	206.7 ± 4	204.5 ± 6	199.7 ± 5	189.1 ± 8
sORP (mV/10^6^ sperm/mL) Normalization	2.08/10^6^	2.43/10^6^	2.40/10^6^	2.35/10^6^	2.22/10^6^

## Data Availability

Original data are available on request.

## Supplementary Materials

The following supporting information can be downloaded at: https://www.mdpi.com/article/10.3390/life14111455/s1, Text SI-1: Value of elements found through EDX energy-dispersive X-ray; Text SI-2: Image of spermatozoa that showed a green fluorescence according to TUNEL test; Text SI-3: Images of spermatozoa that showed a positivity to HSP70 and MTs, after exposure to TiO_2_-NPs; Text SI-4: Images of spermatozoa that showed a positivity to SHBG, after exposure to TiO_2_-NPs; Text SI-5: Summary table of recorded sperm parameters; Text SI-6: Acrosome integrity of exposed groups.
