# Supplementary material for: Susceptibility of Human Spermatozoa to Titanium Dioxide Nanoparticles: Evaluation of DNA Damage and Biomarkers
Source: Life (Basel). 2024 Nov 9;14(11):1455. doi: 10.3390/life14111455 (PMC11595473; doi:10.3390/life14111455)
Supplement: Supplementary file 1 [file life-14-01455-s001.zip › life-3260173-supplementary.pdf]

Susceptibility of Human Spermatozoa to Titanium Dioxide Nanoparticles: Evaluation of DNA Damage and Biomarkers

Elena Maria Scalisi [1,*], Roberta Pecoraro [1], Agata Scalisi [1], Jessica Dragotto [2], Giovanni Bracchitta [2], Massimo Zimbone [3], Giuliana Impellizzeri [3] and Maria Violetta Brundo [1]

[1] Department of Biological, Geological and Environmental Sciences, University of Catania, 95124 Catania, Italy; roberta.pecoraro@unict.it (R.P.); scaliagata@gmail.com (A.S.); mariavioletta.brundo@unict.it (M.V.B.)
[2] U.O. Fisiopatologia della Riproduzione Umana—Clinica del Mediterraneo, 97100 Ragusa, Italy; jessica.dragotto@gmail.com (J.D.); bracchitta@centroaster.com (G.B.)
[3] CNR-IMM, 95123 Catania, Italy; massimo.zimbone@ct.infn.it (M.Z.); giuliana.impellizzeri@ct.infn.it (G.I.)
* Correspondence: elenamaria.scalisi@unict.it; Tel.: +39-0957306039

SUPPLEMENTARY MATERIALS

SI-1: Value of elements found through EDX energy-dispersive X-ray. All results in weight%

Control Group	C	Si	Al	K	O	Au	Ti	Na	Zn
Mean	22,4	11,43	1,03	1,3	67,4	18,0	0	2,3	1,0
Std. deviation	2,5	2,2	0,41	0,97	1,89	0,75	0	1	0,68

500 ppm Group	C	Si	Al	K	O	Au	Ti	Na	Zn
Mean	20,65	26,93	0,47	3,24	65,28	16,14	2,45	3,81	3,54
Std. deviation	2,3	5,0	0,2	0,8	3,4	4,0	0,3	0,5	0,3

250 ppm Group	C	Si	Al	K	O	Au	Ti	Na	Zn
Mean	11,74	27,38	1,76	5,51	53,23	18,70	2,42	4,93	2,38
Std. deviation	8,0	4,5	0,2	0,7	8,5	2,0	1,0	1,7	0,3

100 ppm Group	C	Si	Al	K	O	Au	Ti	Na	Zn
Mean	23,47	12,14	1,79	2,06	68,72	18,38	2,05	2,94	2,15
Std. deviation	4,0	4,3	0,2	0,7	3,7	1,2	0,3	0,4	0,3

50 ppm Group	C	Si	Al	K	O	Au	Ti	Na	Zn
Mean	8,27	22,23	1,28	3,76	53,26	18,38	1,66	4,22	2,11
Std. deviation	0,42	1,0	0,5	0,7	0,4	1,2	0,4	0,1	0,2

SI-2: Images of spermatozoa that showed a green fluorescence according to TUNEL test

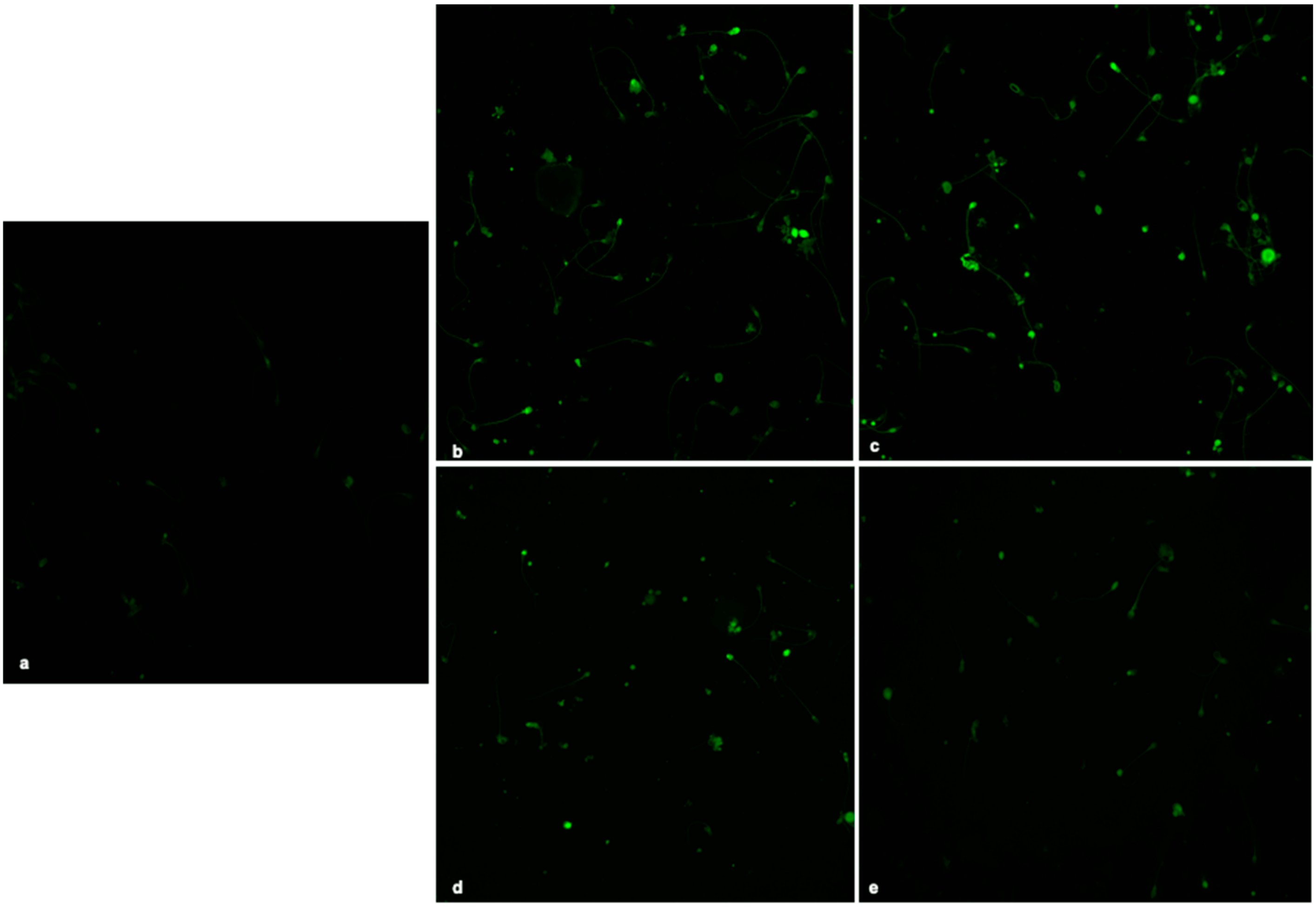

Figure S1. Evaluation of the DNA fragmentation by Tunnel Assay. (**a**) Unexposed spermatozoa, (**b**) Spermatozoa exposed to 500 ppm TiO_2-NPs, (**c**) Spermatozoa exposed to 250 ppm TiO_2-NPs, (**d**) Spermatozoa exposed to 100 ppm TiO_2-NPs, (**e**) Spermatozoa exposed to 50 ppm TiO_2-NPs. 400x.

SI-3: Images of spermatozoa that showed a positivity to HSP70 and MTs, after exposure to TiO_2-NPs

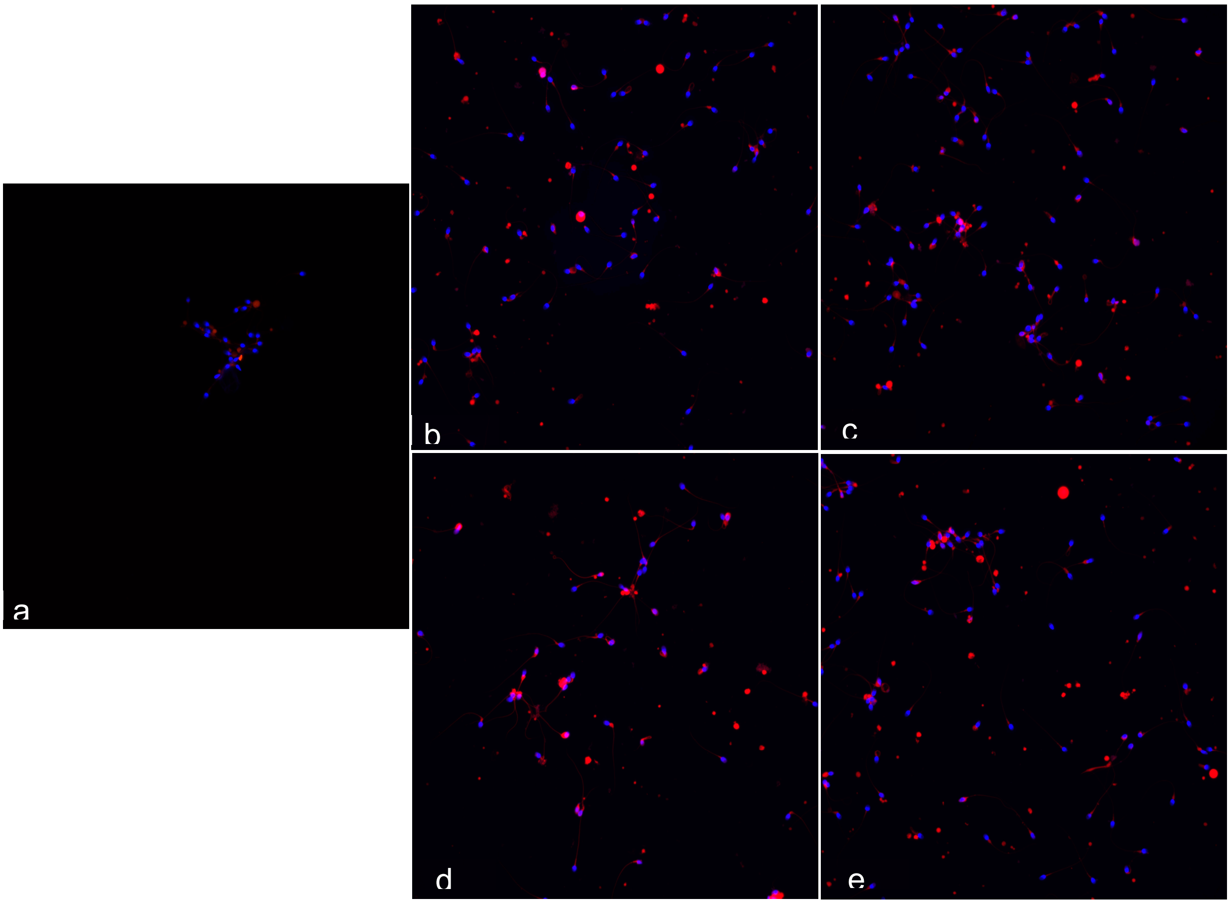

Figure S2. Localization of HSP70 on spermatozoa (**a**) Unexposed spermatozoa, (**b**) Spermatozoa exposed to 500 ppm TiO_2-NPs, (**c**) Spermatozoa exposed to 250 ppm TiO_2-NPs, (**d**) Spermatozoa exposed to 100 ppm TiO_2-NPs, (**e**) Spermatozoa exposed to 50 ppm TiO_2-NPs. 400x.

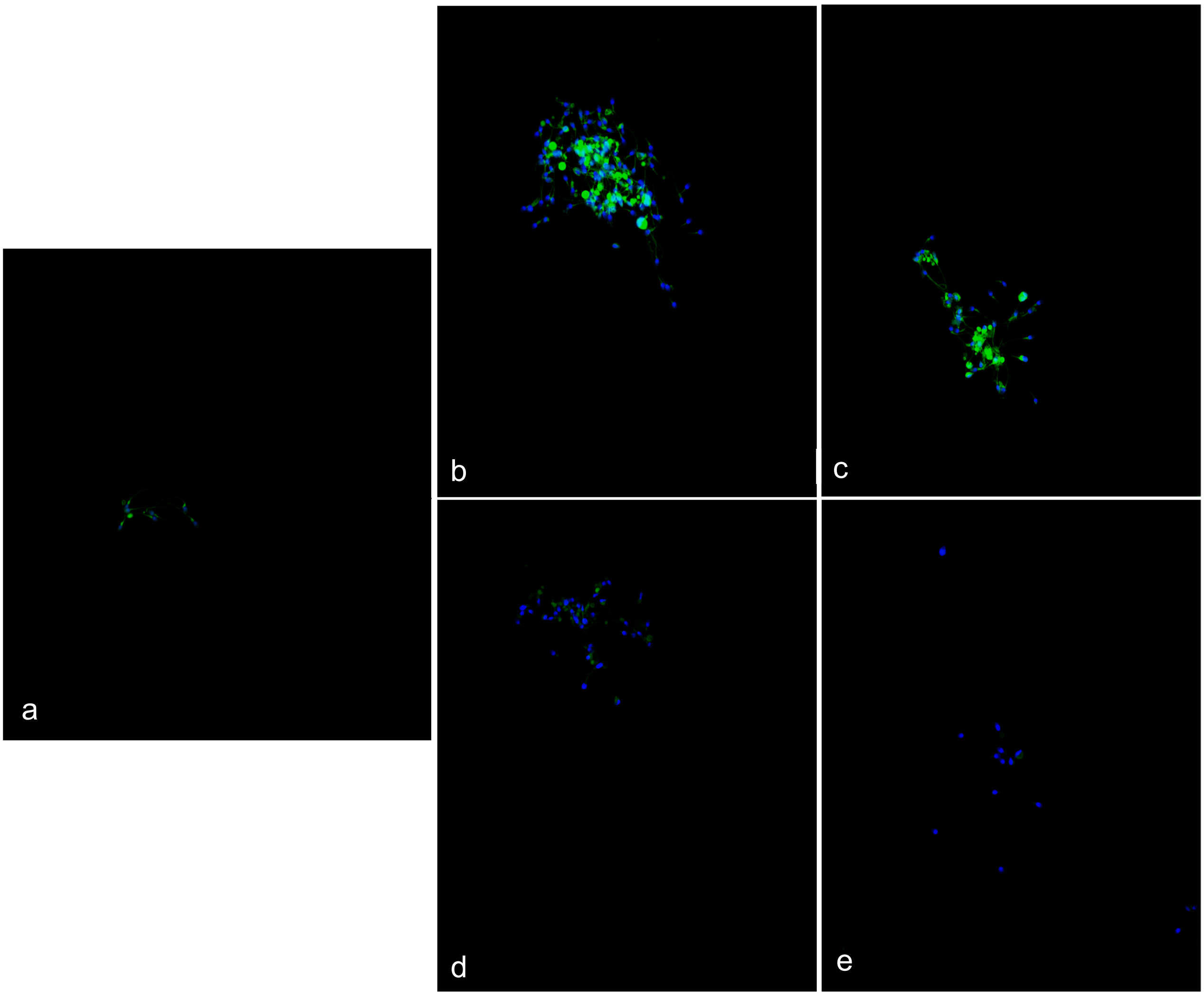

Figure S3. Localization of MTs on spermatozoa (**a**) Unexposed spermatozoa, (**b**) Spermatozoa exposed to 500 ppm TiO_2-NPs, (**c**) Spermatozoa exposed to 250 ppm TiO_2-NPs, (**d**) Spermatozoa exposed to 100 ppm TiO_2-NPs, (**e**) Spermatozoa exposed to 50 ppm TiO_2-NPs. 400x.

SI-4: Images of spermatozoa that showed a positivity to SHBG, after exposure to TiO_2-NPs

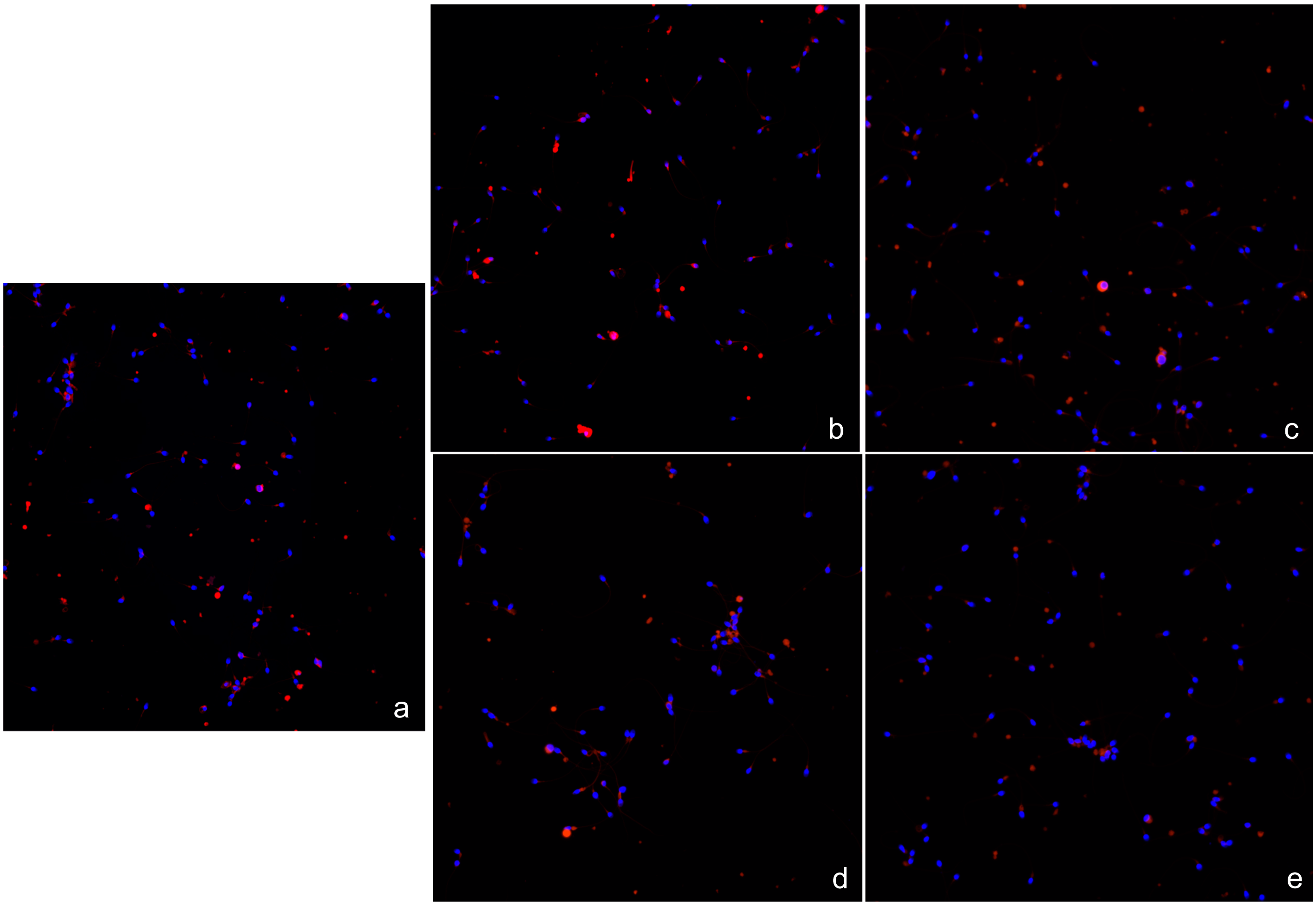

Figure S4. Localization of SHBG on spermatozoa (**a**) Unexposed spermatozoa, (**b**) Spermatozoa exposed to 500 ppm TiO_2-NPs, (**c**) Spermatozoa exposed to 250 ppm TiO_2-NPs, (**d**) Spermatozoa exposed to 100 ppm TiO_2-NPs, (**e**) Spermatozoa exposed to 50 ppm TiO_2-NPs. 400x.

SI-5: Summary table of recorded sperm parameters

Parameters	CTRL	500 ppm	*p*	250 ppm	*p*	100 ppm	*p*	50 ppm	*p*
**DNA fragmentation (%)**	21.6 ± 1.14	41.8±1.30	<0.05*	35.8±1.3	<0.05*	23.8±1.1	0.1	22.6±1.5	0.7
**HSP70 expression (%)**	7.8±0.83	80.2±1.3	<0.05*	77.2±1.3	<0.05*	61±1.6	<0.05*	46.6±1.14	<0.05*
**MTs expression (%)**	4.6±1.14	23.4±2	<0.05*	21.4±1.7	<0.05*	11.8±1.3	<0.05*	6.0±1.6	0.6
**SHBG expression (%)**	46±1.6	75.6±2.4	<0.05*	49.4±2.3	0.1	49.6±1.8	0.07	42.6±1.9	0.1

All data are presented as mean ± standard deviation (SD). A statistically significant difference is indicated with * for p-value<0.05.

SI-6: Acrosome integrity of exposed groups

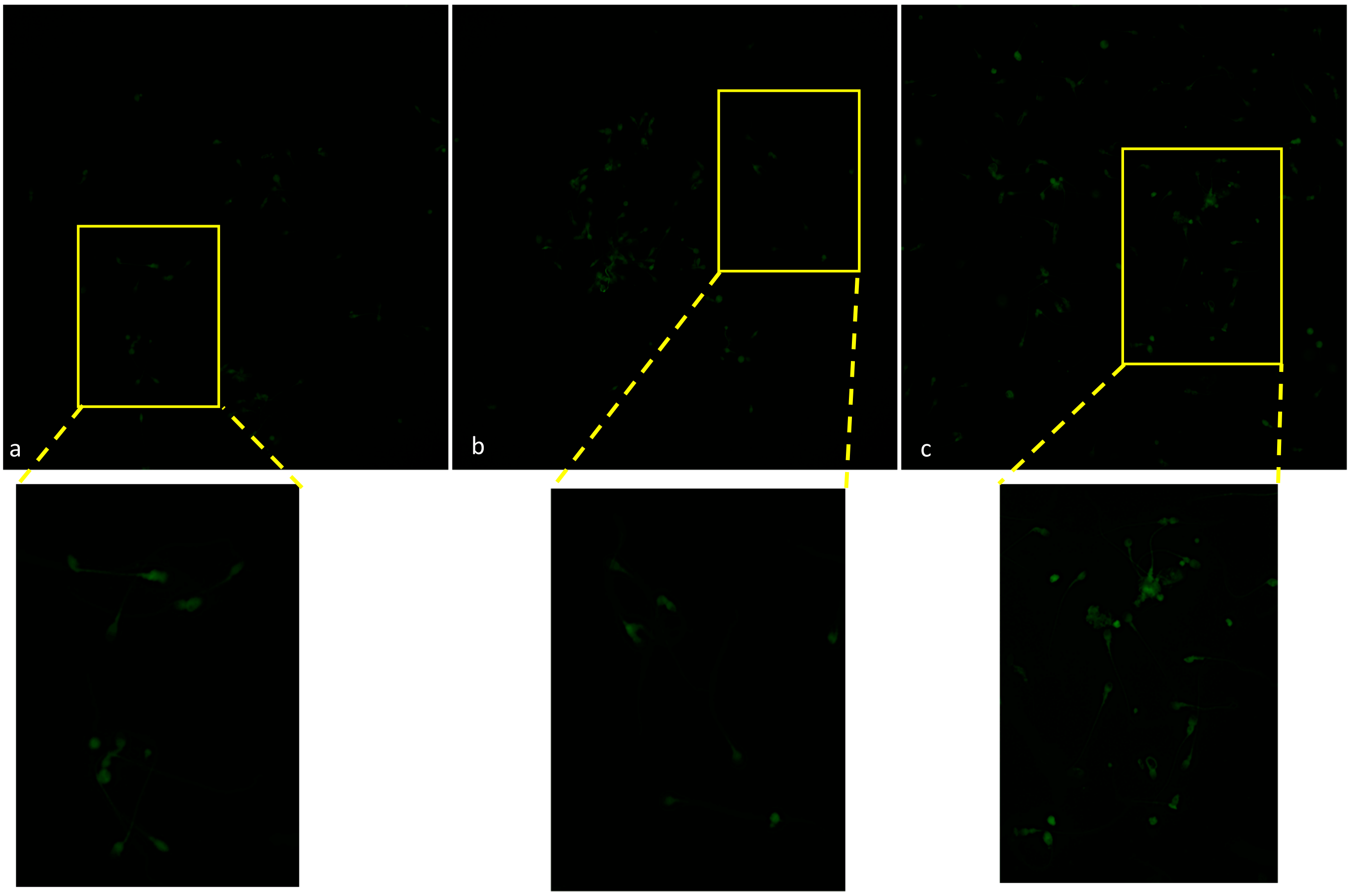

Figure S5. Acrosome integrity assessed by PNA-lectin protocol (**a**) Spermatozoa exposed to 200 ppm TiO_2-NPs, (**b**) Spermatozoa exposed to 100 ppm TiO_2-NPs, (**c**) Spermatozoa exposed to 50 ppm TiO_2-NPs. 400x.
